# A *Drosophila* model relevant to chemotherapy-related cognitive impairment

**DOI:** 10.1038/s41598-023-46616-9

**Published:** 2023-11-07

**Authors:** Matthew Torre, Hassan Bukhari, Vanitha Nithianandam, Camila A. Zanella, Douglas A. Mata, Mel B. Feany

**Affiliations:** 1 Department of Pathology, Brigham and Women’s Hospital and Harvard Medical School, 75 Francis Street, Boston, MA 02115 USA; 2 Foundation Medicine, Inc., Cambridge, MA USA

**Keywords:** Neurological disorders, Diseases of the nervous system

## Abstract

Chemotherapy-related cognitive impairment (CRCI) is a common adverse effect of treatment and is characterized by deficits involving multiple cognitive domains including memory. Despite the significant morbidity of CRCI and the expected increase in cancer survivors over the coming decades, the pathophysiology of CRCI remains incompletely understood, highlighting the need for new model systems to study CRCI. Given the powerful array of genetic approaches and facile high throughput screening ability in *Drosophila*, our goal was to validate a *Drosophila* model relevant to CRCI. We administered the chemotherapeutic agents cisplatin, cyclophosphamide, and doxorubicin to adult *Drosophila*. Neurologic deficits were observed with all tested chemotherapies, with doxorubicin and in particular cisplatin also resulting in memory deficits. We then performed histologic and immunohistochemical analysis of cisplatin-treated *Drosophila* tissue, demonstrating neuropathologic evidence of increased neurodegeneration, DNA damage, and oxidative stress. Thus, our *Drosophila* model relevant to CRCI recapitulates clinical, radiologic, and histologic alterations reported in chemotherapy patients. Our new *Drosophila* model can be used for mechanistic dissection of pathways contributing to CRCI (and chemotherapy-induced neurotoxicity more generally) and pharmacologic screens to identify disease-modifying therapies.

## Introduction

The number of cancer survivors in the United States currently exceeds 18 million^1^ and is expected to increase to 26 million by 2040^2^. Conventional chemotherapy remains a mainstay of cancer treatment, particularly for locally advanced or metastatic tumors. Correspondingly, the global demand for chemotherapy is estimated to rise by over 50% between 2018 and 2040^3^.

Up to 80% of chemotherapy patients develop cognitive deficits^4^, an observation known as chemotherapy-related cognitive impairment (CRCI) or, more colloquially, “chemobrain.” CRCI affects multiple cognitive domains including memory, attention, and executive function^5^. The cognitive deficits observed following chemotherapy treatment are functionally significant and can contribute to reduced ability to function at work or return to work^6^. CRCI may persist for decades after therapy^7^, and some epidemiologic studies have found an association between chemotherapy history and subsequent risk of dementia^8, 9^. Radiologic brain alterations following chemotherapy treatment have been reported, such as decreases in grey matter volume and density, functional/structural connectivity changes, and reduced white matter integrity^10–14^. Risk factors for developing CRCI include advanced age, low cognitive reserve, and certain genetic polymorphisms (e.g. *APOE4* allele)^15, 16^.

Despite the projected increase in cancer survivors and the impact of CRCI on patients’ quality of life and daily functioning^17^, the pathophysiology of CRCI is incompletely understood^18–20^. In vivo CRCI models have been developed in rodents and have implicated mechanisms involving neuroinflammation, proinflammatory cytokines, oxidative stress, DNA damage, direct cytotoxicity on brain cell populations, dysmyelination, and neurogenesis, among others^21–23^. Our goal was to validate a *Drosophila* model relevant to CRCI that can be used as a complementary system to investigate mechanisms contributing to CRCI and chemotherapy-induced neurotoxicity.

*Drosophila melanogaster*, the common fruit fly, is a powerful system to study human disease. Approximately 75% of human disease-causing genes have *Drosophila* orthologs^24^, with most having a conserved cellular function^25^. Compared to mammalian model organisms, *Drosophila* have rapid generation times, shorter life cycles, and are inexpensive to grow and maintain. Importantly, use of *Drosophila* is highly amenable to forward and reverse genetic approaches, including gene editing, fast generation of transgenic lines with tissue- or cell type-specific expression of genes of interest, and high throughput genetic modifier and pharmacologic screens. The strengths of the *Drosophila* model system have resulted in important mechanistic insights into neurodegenerative and neurodevelopmental disorders and forms of iatrogenic neuropathology including chemotherapy-induced peripheral neuropathy^26–29^, highlighting the importance and practicality of a CRCI-relevant *Drosophila* model.

To establish a *Drosophila* model relevant to CRCI, we wanted to determine if chemotherapy-treated flies recapitulate the clinical and neuropathologic features of chemotherapy patients. Thus, we investigated whether chemotherapy-treated flies show neurologic/neurocognitive deficits, evidence of neurodegeneration (corresponding to patients’ neuroradiology changes), and increased DNA damage and oxidative stress, which we have previously described in the brains of chemotherapy patients^30^.

## Results

### Chemotherapy-treated Drosophila show neurologic/neurocognitive deficits

To investigate the effects of chemotherapy administration to flies, adults were aged to 5 days and then transferred to vials containing instant *Drosophila* medium rehydrated with chemotherapy-containing aqueous solution or vehicle (H_2_O) control. Flies were treated with a 3 day regimen of chemotherapy or vehicle, and then transferred back to vials containing standard cornmeal-agar medium and aged to 10, 20, or 30 days for neurologic/neurocognitive testing (climbing assay, taste memory assay) or tissue harvesting. The chemotherapeutic regimens used were cisplatin (10, 100, or 500 μg/ml), cyclophosphamide (10, 100, or 1000 μg/ml), and doxorubicin (10, 100, or 1000 μg/ml). The survival rates of each cohort at 10, 20, and 30 days are provided in Supplemental Table 1.

**Figure 1:**
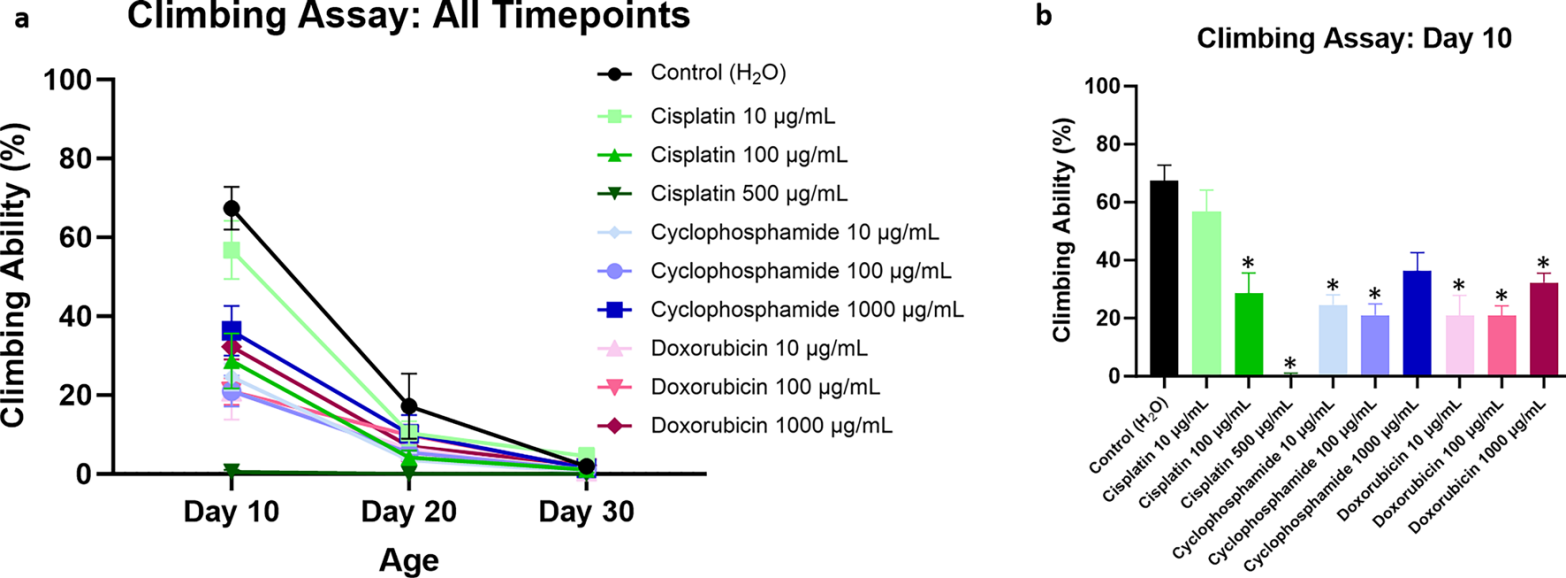
Chemotherapy-treated *Drosophila* show impaired climbing ability, a general neurologic readout. Climbing ability data for all timepoints are shown (**a**). Statistically significant differences are observed at day 10 (**b**). Data are represented as the mean ± SEM. Statistical analysis was performed using the Whitney-Mann U test and Bonferroni correction for multiple testing (corrected *p* value < 0.00555 considered significant). Each cohort consists of n = 6 replicates of 7–12 flies (≥ 53 flies total/cohort) at 10, 20, or 30 days post-eclosion. The genotype is *w*^*1118*^. *p < 0.00555; asterisks are included on the bar graph only.

The climbing assay was performed as a high-throughput screening assay to assess general neurologic function and is based on the negative geotaxis of *Drosophila* (i.e. their intrinsic proclivity to climb upwards). Flies treated with chemotherapy and aged to 10 days showed reduced climbing ability (Fig. 1). Comparisons with the vehicle control group reached statistical significance for the intermediate and high dose cisplatin cohorts (100 and 500 μg/ml), low and intermediate dose cyclophosphamide cohorts (10 and 100 μg/ml), and low, intermediate, and high dose doxorubicin cohorts (10, 100, and 1000 μg/ml). A trend towards reduced climbing ability for the chemotherapy cohorts was also observed at day 20 (Supplemental Fig. 1).

**Figure 2:**
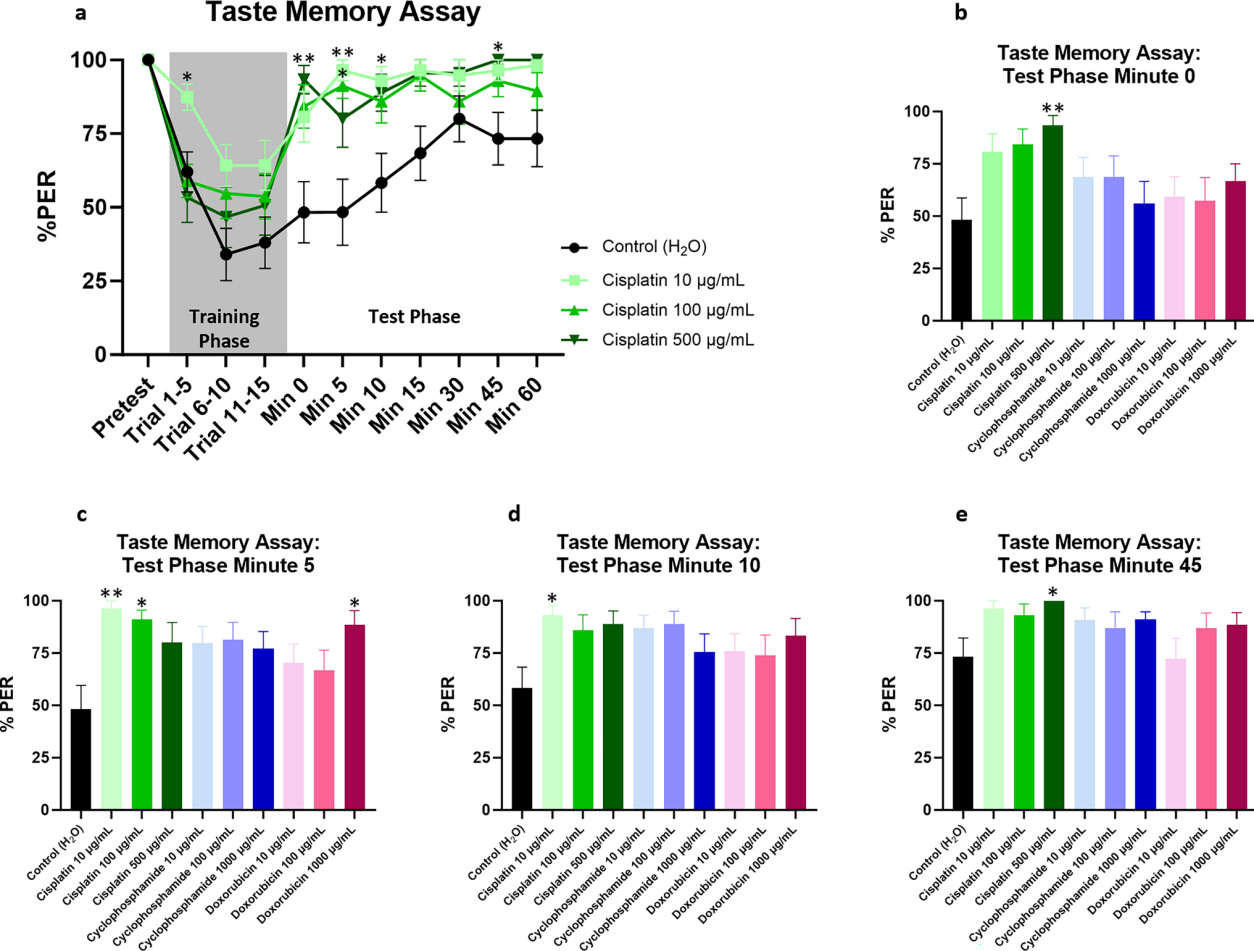
Chemotherapy-treated *Drosophila* have impaired neurocognitive function as assessed by the taste memory assay. Neurocognitive defects are most prominent in cisplatin-treated flies (**a**). Statistically significant differences are observed at minute 0, 5, 10, and 45 of the test phase for cisplatin- and doxorubicin-treated flies (**b**–**e**). Data are represented as the mean ± SEM. Statistical analysis was performed using the repeated measures 2-way ANOVA with Dunnett’s multiple comparison test. Each cohort consists of n ≥ 15 flies at 20 days post-eclosion. The genotype is *w*^*1118*^. *p < 0.05, **p < 0.01. PER, proboscis extension reflex.

The taste memory assay was performed to assess learning and memory (Fig. 2, Supplemental Fig. 2). The taste memory assay is a type of conditioning test and takes advantage of the *Drosophila* proboscis extension reflex (PER). When sucrose solution is presented to their tarsi, *Drosophila* will extend their proboscises to feed. During the taste memory assay, *Drosophila* are trained not to extend their proboscises when their tarsi are exposed to sucrose solution or else their labella are exposed to a bitter, aversive quinine solution. The assay evaluates the ability of flies to learn and remember the aversive stimulus and thus not extend their proboscises. Importantly, flies without an intact PER during the pretest phase of the assay (indicating impaired motor ability to extend the proboscis or attenuated taste sensation) are excluded from the study. During the “Trial 1–5” timepoint of the training phase, the low dose cisplatin cohort (10 μg/ml) had a significantly higher percent of trials with a positive PER compared to the vehicle control group, suggesting a learning defect. Although other chemotherapy cohorts had higher % PER than the vehicle control group at various timepoints during the training phase, these comparisons did not reach statistical significance. At multiple timepoints during the test phase, chemotherapy-treated flies had a higher percent of trials with a positive PER compared to the vehicle control group, suggesting a reduced ability to retain memory of the aversive stimulus. Statistically significant differences were observed for the high dose doxorubicin cohort at minute 5, the high dose cisplatin cohort at minute 0 and 45, the intermediate dose cisplatin cohort at minute 5, and the low dose cisplatin cohort at minute 5 and 10.

Overall, chemotherapy-treated *Drosophila* demonstrated neurologic and neurocognitive deficits as assessed by the climbing assay and taste memory assay, highlighting the relevance of the *Drosophila* model to study CRCI. The deficits were observed with different chemotherapeutic agents (over a range of concentrations) with diverse mechanisms of action but were particularly prominent following cisplatin treatment.

### Cisplatin exposure exacerbates neurodegeneration in Drosophila

**Figure 3:**
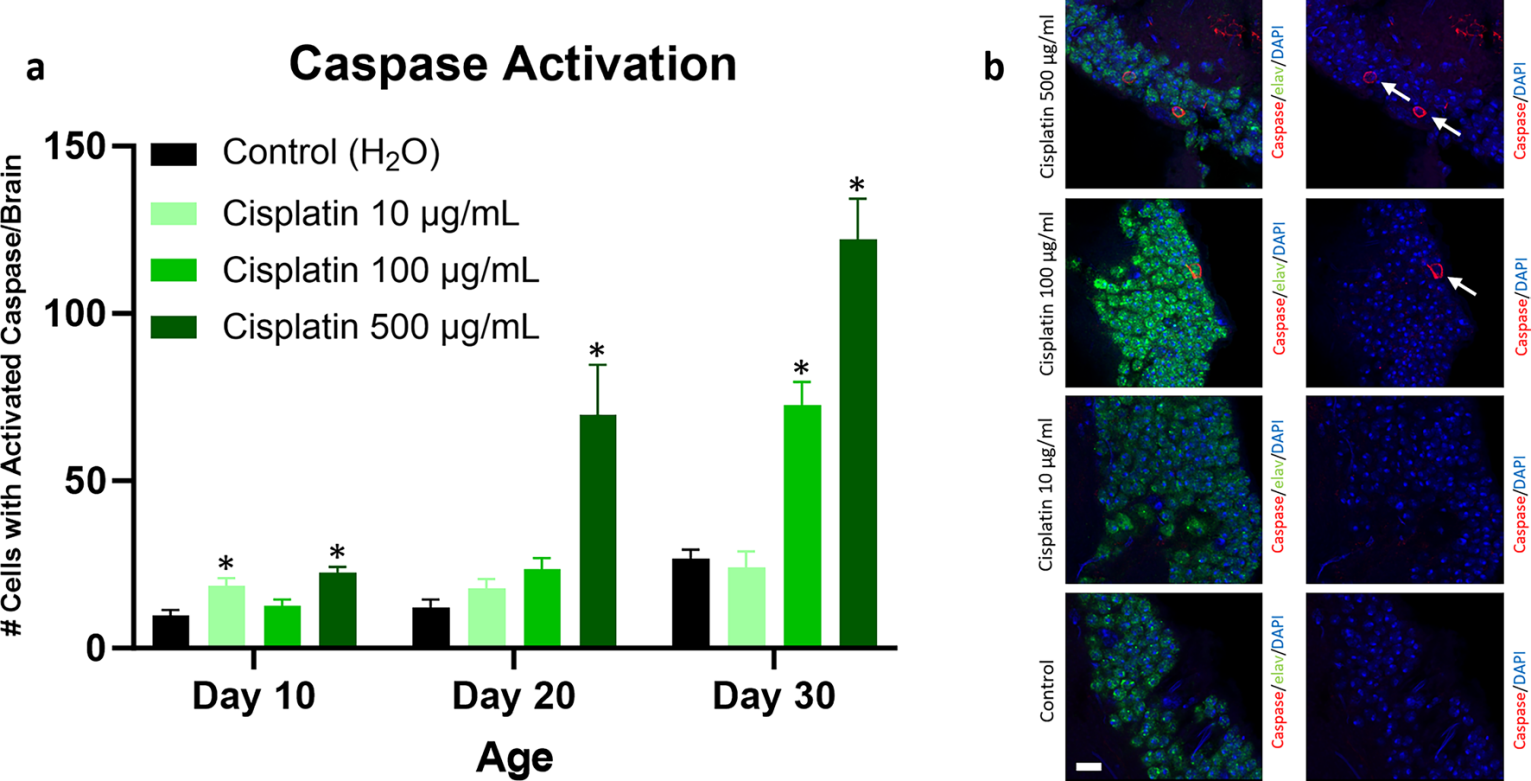
Neurodegeneration is increased in cisplatin-treated *Drosophila*, as assessed by caspase activation. Flies given cisplatin have an increase in the number of brain cells with caspase activation, predominantly elav positive neurons (**a**). Immunofluorescence images of brains from cisplatin and control flies at day 30 are shown (**b**). Arrows highlight neurons showing caspase activation; scale bar, 5 μm. Data are represented as the mean ± SEM. Statistical analysis was performed using the Whitney-Mann U test and Bonferroni correction for multiple testing (corrected *p* value < 0.0166 considered significant). N = 6 for all cohorts at 10, 20, and 30 days post-eclosion. The genotype is *UAS-CD8-PARP-Venus*/*da-GAL4*. *p < 0.0166.

**Figure 4:**
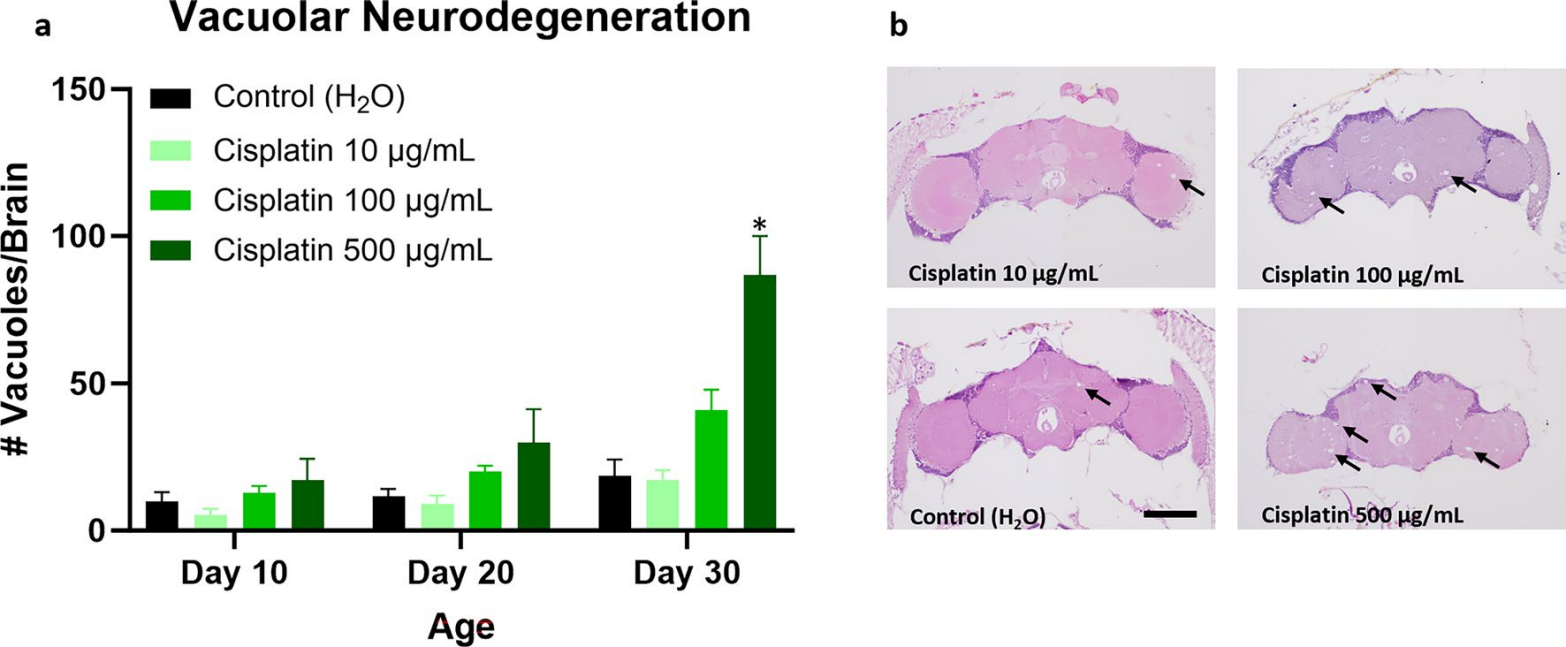
Neurodegeneration is increased in cisplatin-treated *Drosophila*, as assessed by brain vacuoles. Flies given cisplatin have an increase in the number of brain vacuoles (**a**). Hematoxylin and eosin images of brains from cisplatin and control flies at day 30 are shown (**b**). Arrows highlight representative vacuoles; scale bar, 100 μm. Data are represented as the mean ± SEM. Statistical analysis was performed using the Whitney-Mann U test and Bonferroni correction for multiple testing (corrected *p* value < 0.0166 considered significant). N = 6 for all cohorts at 10, 20, and 30 days post-eclosion. The genotype is *w*^*1118*^. *p < 0.0166.

Because treatment with cisplatin resulted in the most severe neurologic and neurocognitive deficits, we decided to focus our subsequent tissue analysis on flies given cisplatin. We assessed neurodegeneration in *Drosophila* by quantifying the number of cells with caspase activation and the number of brain vacuoles. We used *da-GAL4* to drive ubiquitous expression of transgenic caspase reporter *UAS-CD8-PARP-Venus*. The *UAS-CD8-PARP-Venus* reporter flies contain a transgenic construct composed of the extracellular and transmembrane domains of mouse CD8 fused to a 40 amino acid sequence from human PARP that includes the caspase cleavage site^31^. Activated endogenous *Drosophila* caspases cleave human PARP at the caspase cleavage site. Caspase activation is then assessed using an antibody that specifically binds to cleaved human PARP^32–34^. We found significantly increased numbers of cells with caspase activation in the high dose cisplatin cohort at day 10, 20, and 30, the intermediate dose cisplatin cohort at day 30, and the low dose cisplatin cohort at day 10 compared to the vehicle control cohort (Fig. 3). The brain cells with activated caspase were predominantly neurons (demonstrated by double labeling immunofluorescence with elav, a neuron specific marker) but also included glia. Flies treated with high dose cisplatin also had significantly increased brain vacuoles compared to vehicle control at day 30 (Fig. 4). Vacuoles were seen in optic lobe and central brain including mushroom body. The formation of brain vacuoles is frequently associated with neurodegeneration in *Drosophila*^35–38^. In summary, exposure to chemotherapeutic agent cisplatin was associated with elevated indicators of neurodegeneration, including caspase activation and brain vacuoles.

### DNA damage is elevated after cisplatin exposure

**Figure 5:**
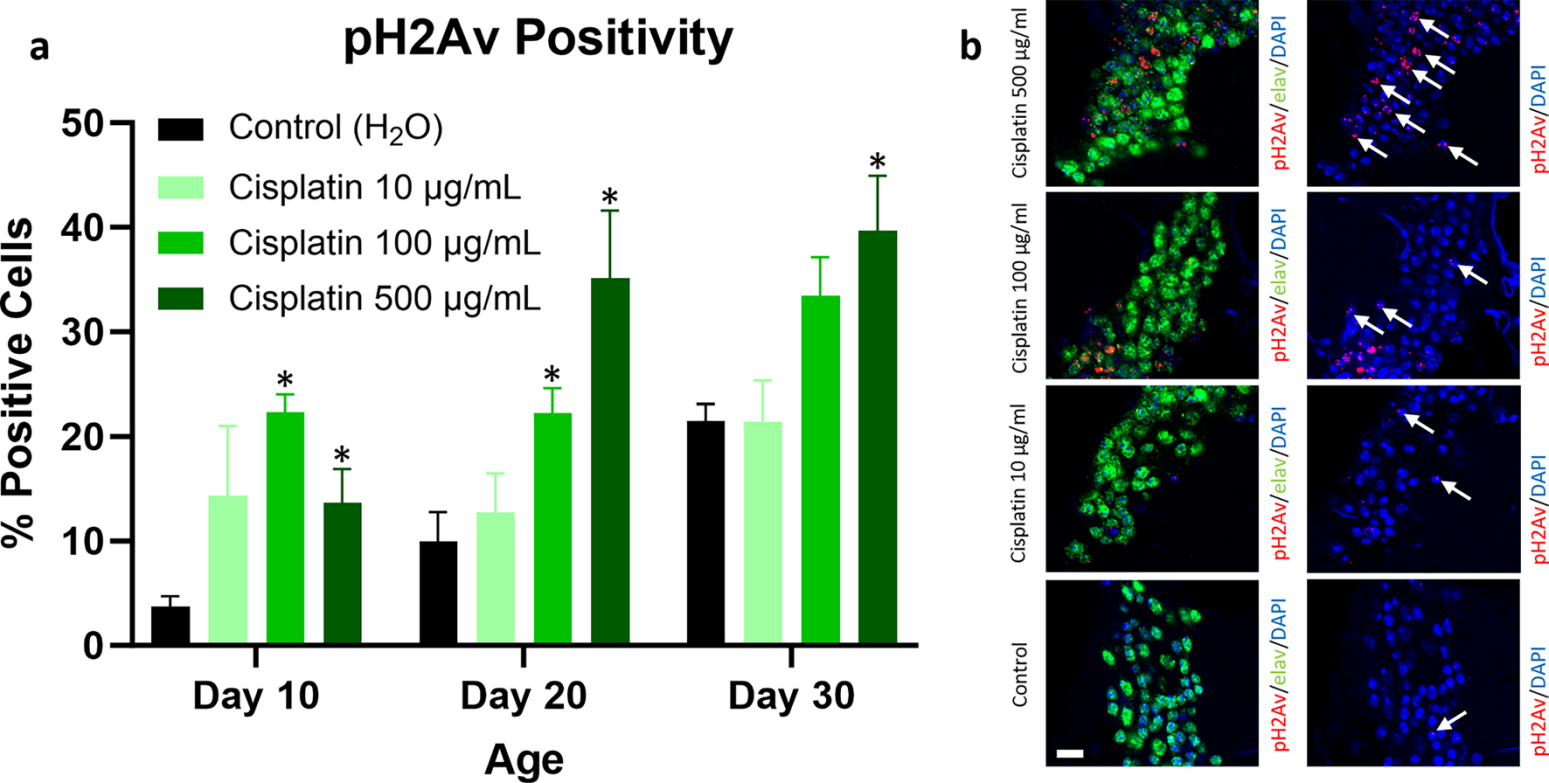
DNA damage is increased in cisplatin-treated *Drosophila*. Flies given cisplatin have an increase in the percent of pH2Av positive cells, predominantly elav positive neurons, within an anatomically consistent region of the mushroom body (**a**). Immunofluorescence images of brains from cisplatin and control flies at day 30 are shown (**b**). Arrows highlight representative neurons with pH2Av punctate staining; scale bar, 5 μm. Data are represented as the mean ± SEM. Statistical analysis was performed using the Whitney-Mann U test and Bonferroni correction for multiple testing (corrected *p* value < 0.0166 considered significant). N = 6 for all cohorts at 10, 20, and 30 days post-eclosion. The genotype is *w*^*1118*^. *p < 0.0166.

DNA damage was assessed by quantifying the percent of cells showing phosphorylation of serine 137 of histone variant H2Av (pH2Av), a marker of DNA double-strand breaks. We focused our quantification on an anatomically consistent region of the mushroom body, an area of the *Drosophila* brain that is important for learning and memory^39, 40^. A significantly increased percent of pH2Av positive cells was seen in the high dose cisplatin cohort at day 10, 20, and 30 and the intermediate dose cisplatin cohort at day 10 and 20 compared to the vehicle control cohort (Fig. 5). The pH2Av positive brain cells were predominantly neurons (supported by double labeling immunofluorescence with the neuronal marker elav) but also included glia. Thus, chemotherapy exposure resulted in increased DNA damage in the brain.

### Oxidative stress is elevated after cisplatin exposure

**Figure 6:**
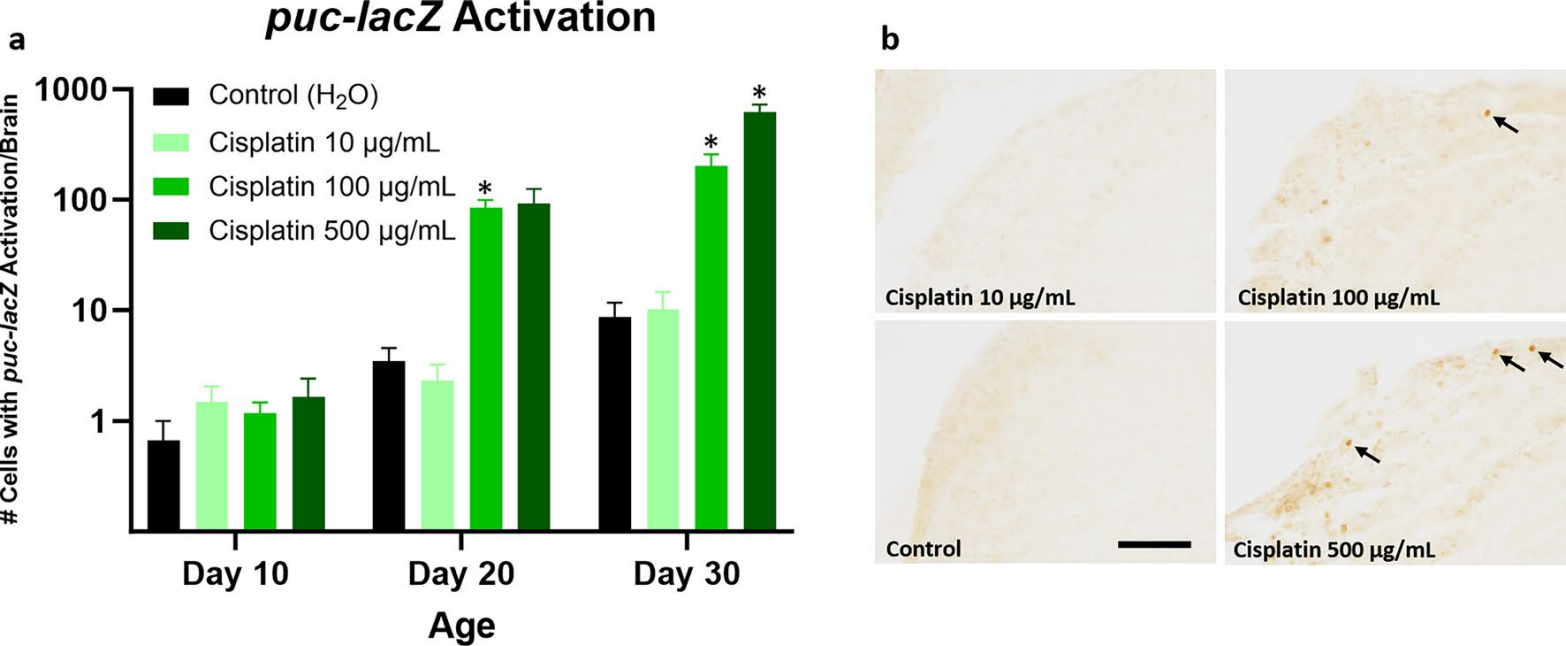
Oxidative stress is increased in cisplatin-treated *Drosophila*, as assessed by *puc-lacZ* activation. Flies given cisplatin have increased brain cells with *puc-lacZ* reporter activation, indicated by β-galactosidase immunopositivity (**a**). Immunohistochemical images of brains from cisplatin and control flies at day 30 are shown (**b**). Arrows highlight representative cells with *puc-lacZ* activation; scale bar, 20 μm. Data are represented as the mean ± SEM. Statistical analysis was performed using the Whitney-Mann U test and Bonferroni correction for multiple testing (corrected *p* value < 0.0166 considered significant). N = 6 for all cohorts at 10, 20, and 30 days post-eclosion. The genotype is *puc-lacZ*/ + . *p < 0.0166.

**Figure 7:**
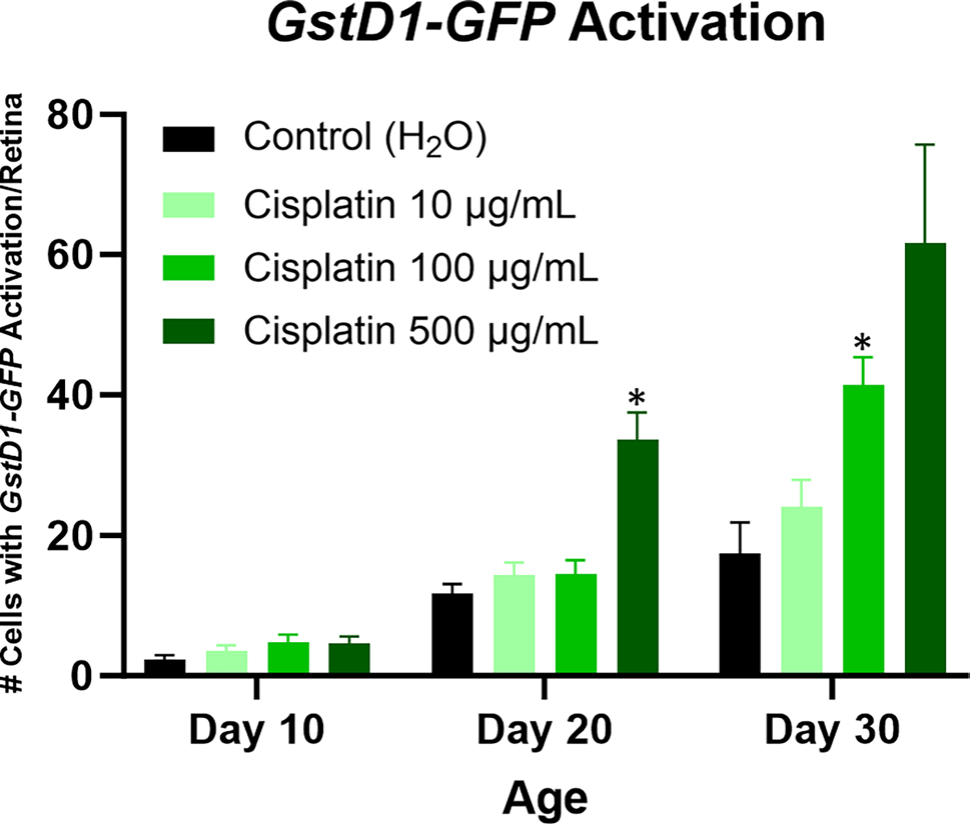
Oxidative stress is increased in cisplatin-treated *Drosophila*, as assessed by *GstD1-GFP* activation. Flies given cisplatin demonstrate an increased number of retinal cells with *GstD1-GFP* reporter activation. Data are represented as the mean ± SEM. Statistical analysis was performed using the Whitney-Mann U test and Bonferroni correction for multiple testing (corrected *p* value < 0.0166 considered significant). N = 6 for all cohorts at 10, 20, and 30 days post-eclosion. The genotype is *GstD1-GFP*/ + . *p < 0.0166.

Oxidative stress was assessed using the *puckered-lacZ* (*puc-lacZ*) and *GstD1-GFP* reporter systems. The *puc* gene encodes a phosphatase that is both a downstream target and a negative regulator of the JNK signaling pathway, which is activated in response to oxidative stress^41–43^. Activation of the *puc-lacZ* reporter can be detected by β-galactosidase immunopositivity. We found that flies treated with cisplatin had increased numbers of brain cells with *puc-lacZ* activation compared to flies treated with vehicle control, reaching statistical significance in the high dose cisplatin cohort at day 30 and the intermediate dose cisplatin cohort at day 20 and 30 (Fig. 6). Double labeling immunofluorescence with elav (data not shown) demonstrated *puc-lacZ* activation in neurons, as previously reported^41^. *GstD1* is an oxidative stress response gene and encodes glutathione S-transferase D1^44^. Activation of the *GstD1-GFP* reporter^45^ can be detected by GFP immunopositivity. Preliminary immunofluorescence data suggested that differential activation of *GstD1-GFP* was more prominent in the retina than the brain following chemotherapy. Subsequent analysis confirmed that cisplatin-treated flies had increased cells in the retina with *GstD1-GFP* activation compared to vehicle control, reaching statistical significance in the high dose cisplatin cohort at day 20 and the intermediate dose cisplatin cohort at day 30 (Fig. 7). Representative *GstD1-GFP* images are shown in Supplemental Fig. 3. In summary, chemotherapy exposure was associated with elevated markers of oxidative stress.

## Discussion

CRCI is a clinically important and functionally significant adverse effect of chemotherapy observed in up to 80% of patients^4^. However, development of effective CRCI disease-modifying therapies is hindered by a limited understanding of the pathophysiology of CRCI. Thus, new approaches to investigate CRCI are needed. We present a working *Drosophila* model relevant to CRCI (and chemotherapy-induced neurotoxicity more generally) that recapitulates clinical and neuropathologic features described in chemotherapy patients.

We used the climbing assay as a high throughput screening test for general neurologic function. As a general neurologic readout, the climbing assay reflects the status of the CNS, PNS, and neuromuscular system. We observed neurologic deficits in our *Drosophila* model following treatment with different chemotherapeutic agents (with diverse mechanisms of action) at multiple concentrations. Cisplatin, cyclophosphamide, and doxorubicin are commonly administered to breast cancer patients, the patient population most studied in CRCI^46^, and are among a group of chemotherapies whose use is associated with CRCI^23, 47^. The concentrations of chemotherapy that we tested were informed by previously published doses administered to *Drosophila*^48–57^. Our concentration of cisplatin ranged from 10 to 500 μg/ml, and a prior study reported that a cisplatin regimen of 100 μg/ml resulted in a whole *Drosophila* body concentration comparable to that used in human patients and rodent models of cisplatin-induced neurotoxicity^48^.

We then used the taste memory assay to evaluate neurocognitive function, making the assay particularly well-suited to model CRCI. Importantly, unlike other methods of assessing cognition in *Drosophila* such as the olfactory conditioning assay or aversive phototaxis suppression assay, the taste memory assay is not dependent on locomotor ability. Chemotherapy-treated flies showed a consistent trend towards worse performance during the test phase of the taste memory assay (Fig. 2 and Supplemental Fig. 2), becoming statistically significant at multiple timepoints. Memory deficits were most prominent in cisplatin-treated flies. Assay noise may have contributed to fluctuations in performance during the training and test phases of the taste memory assay and reduced the assay’s sensitivity to detect more subtle cognitive defects. Thus, in follow-up studies, we would recommend increasing the cohort sizes for the taste memory assay to reduce noise.

Because cisplatin resulted in the most marked neurocognitive deficits in our model, we decided to focus our neuropathologic evaluation on cisplatin-treated flies. Given that chemotherapy patients show radiologic changes in the brain including reductions in grey matter volume and density^10, 11^, we were interested in assessing neurodegeneration in chemotherapy-treated *Drosophila*. We demonstrated an increase in the number of cells with caspase activation after cisplatin exposure. Our finding is in keeping with previous studies showing increased apoptosis and cell death in vulnerable brain cell populations following chemotherapy in in vivo and in vitro models^58–60^. We observed that the number of cells with activated caspase was elevated acutely after cisplatin treatment (i.e. at day 10) and remained persistently elevated at day 20 and 30. Thus, chemotherapy appears to induce long term changes that promote ongoing caspase activation with age. This is supported by our vacuolar neurodegeneration data, which showed an increase in brain vacuoles in the high dose cisplatin group at day 30 but not at earlier timepoints. The presence of brain vacuoles in the cortex and neuropil plausibly reflects cell death and loss of axons^35, 61, 62^. Caspase activation may be more sensitive/specific than vacuole formation for detecting neurodegeneration, possibly accounting for differences between these two assays. Overall, our *Drosophila* model showed increased neurodegeneration by both the number of cells with caspase activation in the brain and by the number of brain vacuoles.

We have previously reported that chemotherapy patients have elevated DNA damage and oxidative stress in frontal lobe cortical neurons compared to control patients^30^. We thus wanted to assess whether these pathways were upregulated in our CRCI-relevant *Drosophila* model. Flies treated with cisplatin showed increased DNA damage, indicated by pH2Av positivity, compared to vehicle control. Similar to our observations with caspase activation, DNA damage increased acutely after cisplatin treatment and continued to be elevated long after cessation of chemotherapy. Oxidative stress (indicated by *puc-lacZ* and *GstD1-GFP* reporter activation in the brain and retina, respectively) was elevated in cisplatin-treated flies compared to vehicle control, but statistically significant differences were observed only at day 20 and 30. However, we cannot exclude the possibility that other markers of oxidative stress may be elevated more acutely.

Differences that we observed in our *Drosophila* model in the timing of DNA damage and oxidative stress may implicate certain biological pathways in chemotherapy-induced neurotoxicity in the brain. Cisplatin is known to damage both nuclear and mitochondrial DNA^63^, and our data suggest that DNA damage occurs early after chemotherapy exposure. Cisplatin-induced DNA damage promotes the production of mitochondrial derived reactive oxygen species (ROS)^64^ that over time can result in measurable increases in oxidative stress. These ROS may cause additional DNA damage, creating a positive feedback loop that contributes to persistently elevated DNA damage, oxidative stress, and caspase activation long after the initial chemotherapy insult is removed. It is compelling to speculate whether cellular senescence arising in the setting of chemotherapy may have a role in these persistent changes.

Our study has a few limitations. Firstly, while the data implicate promising molecular pathways in CRCI, more mechanistic analysis is needed to determine if the neurotoxic effects of chemotherapy are due to its direct action on brain cell populations or through an indirect mechanism or a combination of the two. Secondly, while we demonstrate that multiple chemotherapeutic agents cause neurologic/neurocognitive deficits in *Drosophila*, our tissue analysis focused on cisplatin since it resulted in the most significant decrement to neurocognitive function. Although we anticipate that the use of *Drosophila* will accelerate the mechanistic understanding of CRCI, we should emphasize that the pathophysiology of CRCI is multifactorial and that *Drosophila* may better recapitulate some mechanisms than others. Blood brain barrier permeability to chemotherapeutic agents, alterations in neurogenesis, and microglial activation likely contribute to CRCI, but additional studies are needed to determine whether these mechanisms can be appropriately modeled in *Drosophila*.

In summary, we present a novel *Drosophila* model relevant to CRCI. Our *Drosophila* model demonstrates neurologic/neurocognitive deficits, increased neurodegeneration, and elevated DNA damage and oxidative stress, recapitulating the clinical and neuropathologic features of chemotherapy patients. Our *Drosophila* model may thus serve as a complementary system to existing rodent CRCI models and take advantage of powerful *Drosophila* genetic tools, high throughput screening, low operational cost, and short lifespan. Our model can be easily adapted to test other chemotherapeutic drugs at different concentrations, timepoints, and combinations. We are also interested in dissecting phenotypic differences by sex and investigating how vulnerability to chemotherapy-induced injury may be mediated by sex. Future *Drosophila* CRCI studies can leverage forward and reverse genetic approaches to decipher molecular pathways contributing to CRCI, identify specific cell types that are particularly vulnerable to chemotherapy-induced injury, use pharmacologic screens to identify agents that may ameliorate CRCI, and investigate the mechanistic interactions between CRCI and tau pathology, synuclein pathology, and cellular senescence, among other pathways relevant to neurodegeneration.

## Materials and methods

### *Drosophila* stocks and genetics

All *Drosophila* crosses were performed at 25°C. The following stocks were obtained from the Bloomington *Drosophila* Stock Center (NIH P40OD018537) at Indiana University, Bloomington, IN: *w*^*1118*^, *da-GAL4*, and *puc*^*E69*^ (*puc-lacZ).* Darren Williams (Kings College, London, United Kingdom) provided the *UAS-CD8-PARP-Venus* stock. Dirk Bohmann (University of Rochester Medical Center, Rochester, NY) provided the *GstD1-GFP* stock. Male and female flies were used for all experiments.

### Chemotherapy regimens and maintenance

Flies of the appropriate genotype were aged to 5 days post-eclosion in vials containing standard cornmeal-agar medium and then transferred to vials of instant medium (Carolina Biological; Burlington, NC) rehydrated with chemotherapy-containing aqueous solution or with vehicle (H_2_O) control (Milli Q water, MilliporeSigma; Burlington, MA). Flies were placed on a 3 day regimen of rehydrated instant medium, which was replaced daily. Following this 3 day regimen, flies were transferred back to vials containing standard cornmeal-agar medium and then aged to 10, 20, or 30 days post-eclosion for testing and/or harvesting for histologic studies. Chemotherapeutic agents cisplatin (10, 100, and 500 μg/ml), doxorubicin (10, 100, and 1000 μg/ml), and cyclophosphamide (10, 100, and 1000 μg/ml) were dissolved in Milli Q water. Chemotherapeutic agents were purchased from MilliporeSigma. Drug concentrations for each chemotherapeutic agent were similar to those used in prior *Drosophila* studies^48–57^. Aging was performed at 25 °C.

### Climbing assay

Flies underwent the climbing assay at 10, 20, or 30 days post-eclosion. Individual flies were tested at one timepoint only. The climbing assay was performed based on standard protocols^36^. For each timepoint and treatment cohort, 7–12 flies were placed in individual empty vials for a total of 6 vials. At least 53 flies were tested in each group. Vials were gently tapped to bring the flies to the bottom of the vial, and the percent of flies that climbed above 5 cm within 10 s was recorded. The climbing assay was repeated 3 times for each vial of flies and averaged. An approximately equal proportion of males to females was used for all cohorts.

### Taste memory assay

The taste memory assay was performed on chemotherapy or vehicle control treated flies at 20 days post-eclosion and based on standard protocols with minor modifications^65–67^. Flies were starved for 24 h prior to testing by being transferred to empty vials with Milli Q water-soaked Kimwipes (Kimberly-Clark; Franklin, MA). Flies were then briefly anesthetized with CO_2_, fixed to glass slides with nail polish, and moved to a 25 °C humidified incubator for a 3 h recovery period. The slides were then vertically mounted and placed under a dissecting microscope to observe the fly behavior. Flies were satiated with purified water, and flies that are not satiated after 5 min were excluded from the study.

The taste memory assay consists of 3 phases: a pretest phase, a training phase, and a test phase. During the pretest phase, a 500mM sucrose solution was presented to the fly tarsi to confirm an intact proboscis extension reflex (PER). Flies with negative PER were excluded from further study. The training phase consists of 15 trials. During these trials, fly tarsi were exposed to 500 mM sucrose solution, but when the proboscis extended, a bitter, aversive solution (50 mM quinine) was presented to the labellum. Flies were allowed to drink the quinine solution for up to 2 s or until they retracted their proboscises. Between trials, the tarsi and labellum were washed with water, and the flies were allowed to drink fresh water to satiation. The training trials were binned into 3 groups of 5 trials (trials 1–5, 6–10, and 11–15). During the test phase, the 500 mM sucrose solution was presented to the tarsi at multiple time intervals (0, 5, 10, 15, 30, 45, and 60 min) but the labellum was not exposed to quinine solution if the proboscis extended. At each test timepoint, flies underwent 3 trials with a 10 s interval between trials. The percent of trials with a positive PER was recorded for each fly at each timepoint. After each test timepoint, the tarsi were washed with water, and the flies were allowed to drink fresh water to satiation. At the end of the test phase, flies were given sucrose solution to confirm an intact PER, and flies with negative PER were excluded from the analysis. Sucrose and quinine solutions were presented to the flies as droplets at the end of 1 mL syringes with 21 gauge needles. At least 15 flies were tested per cohort, with an approximately equal proportion of males to females.

### Histology, immunohistochemistry, and immunofluorescence

Flies were harvested at 10, 20, or 30 days post-eclosion and fixed in formalin. Fly heads were embedded in paraffin and serially sectioned through the entire brain and retina (either 2 or 4 µm thick sections) and processed through a series of xylenes, ethanol, and water. Vacuolar neurodegeneration was evaluated in 2 µm brain sections stained with hematoxylin and eosin (H&E). For the immunostaining studies (4 µm sections), incubation with the following primary antibodies was performed following routine heat antigen retrieval (10 mM sodium citrate buffer, pH 6.0): anti-cleaved PARP (Abcam, E51, Cambridge, MA; 1:50,000 for immunohistochemistry (IHC), 1:500 for immunofluorescence (IF)), anti-β-galactosidase (Promega, Z3783, Madison, WI; 1:500 for IHC, 1:100 for IF), anti-GFP (NeuroMab, N86/8, Davis, CA; 1:500 for IHC and IF), anti-pH2Av (Rockland, 600–401-914, Pottstown, PA; 1:2500 for IHC, 1:1000 for IF), and anti-elav (Developmental Studies Hybridoma Bank, clones 9F8A9 and 7E8A10, Iowa City, IA; 1:5 (9F8A9) or 1:50 (7E8A10) for IF). For IHC, slides were then incubated with biotinylated secondary antibodies (Southern Biotech, Birmingham, AL; 1:200), and the markers were visualized with the avidin–biotin complex (ABC) detection system (Vector Laboratories, Burlingame, CA) and 3,3′-diaminobenzidine (DAB). pH2Av IHC slides were counterstained with hematoxylin. For IF, slides were incubated with Alexa Fluor-conjugated secondary antibodies (Invitrogen, Alexa 488, Alexa 555, Carlsbad, CA; 1:200) and mounted with 4',6-diamidino-2-phenylindole (DAPI)-containing Fluoromount medium (Southern Biotech).

For quantification of vacuolar neurodegeneration, caspase activation, and *puc-lacZ* activation, the number of vacuoles or the number of immunopositive cells throughout the entire brain was counted per fly, and the average number of vacuoles or immunopositive cells per brain was averaged for each cohort (n = 6 per cohort). We quantified *GstD1-GFP* activation by counting the number of GFP immunopositive cells throughout the entire retina bilaterally in each fly. The average number of immunopositive cells in the bilateral retina was averaged for each cohort (n = 6 per cohort). pH2Av quantification was performed by calculating the percent of pH2Av-positive cells within a 1000 × high power field of an anatomically consistent region of mushroom body containing Kenyon cells. The percent of pH2Av-positive cells was averaged for each cohort (n = 6 per cohort). Equal numbers of males and females were evaluated in each cohort for the histology and immunohistochemistry studies. H&E and IHC slides were analyzed using a Nikon Eclipse E600 microscope with SPOT software. H&E photos were taken with an Olympus DP25 camera, and IF photos were taken with a Zeiss LSM 800 confocal microscope.

### Statistical analysis

For the climbing assay, chemotherapy cohorts were compared to the H_2_O vehicle control cohort using the Whitney-Mann U test and Bonferroni correction for multiple testing. A two-sided study-wide α level of 0.05 was considered significant. Since there were 9 comparisons (3 different chemotherapeutic agents at 3 concentrations compared to control) per timepoint, a corrected *p* value threshold of 0.00555 was used to determine significance. For the taste memory assay, the chemotherapy cohorts were compared to the H_2_O vehicle control cohort using a repeated measures 2-way ANOVA with Dunnett’s multiple comparison test. *P* values < 0.05 were considered significant. For the histology and immunohistochemistry studies, chemotherapy cohorts were compared to the H_2_O vehicle control cohort using the Whitney-Mann U test and Bonferroni correction for multiple testing. With a 2-sided study-wide α level of 0.05 for significance and 3 comparisons per timepoint per study (3 concentrations of cisplatin compared to control), a corrected *p* value threshold of 0.0166 was used to determine significance.

### Supplementary Information

Supplementary Information.

## Data Availability

All study data are included in the article.
