# Supplementary material for: A Drosophila model relevant to chemotherapy-related cognitive impairment
Source: Sci Rep. 2023 Nov 7;13:19290. doi: 10.1038/s41598-023-46616-9 (PMC10630312; doi:10.1038/s41598-023-46616-9)
Supplement: Supplementary file 1 — Supplementary Information. [file 41598_2023_46616_MOESM1_ESM.pdf]

Supplemental Figure 1

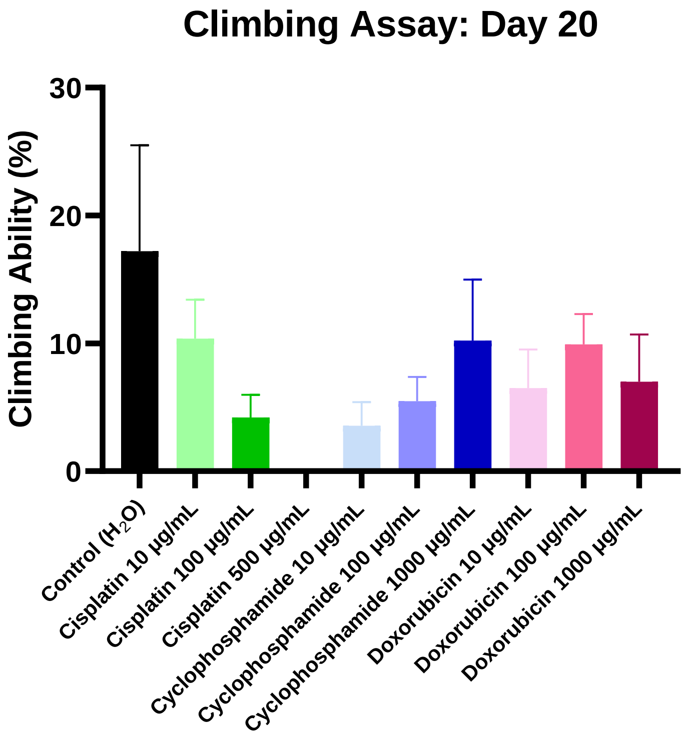

Chemotherapy-treated *Drosophila* show a trend towards impaired climbing ability, a general neurologic readout, at day 20. Data are represented as the mean ± SEM. Statistical analysis was performed using the Whitney-Mann U test and Bonferroni correction for multiple testing (corrected p value <0.00555 considered significant). Each cohort consists of n=6 replicates of 7-12 flies (≥53 flies total/cohort). The genotype is w^{1118}. *p<0.00555

Supplemental Figure 2

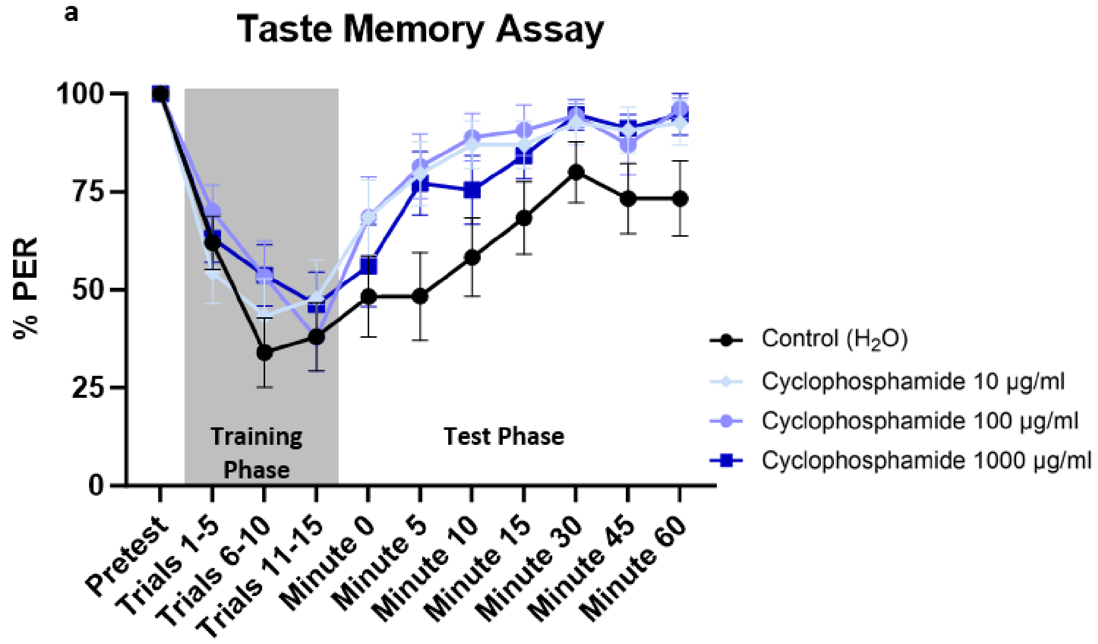

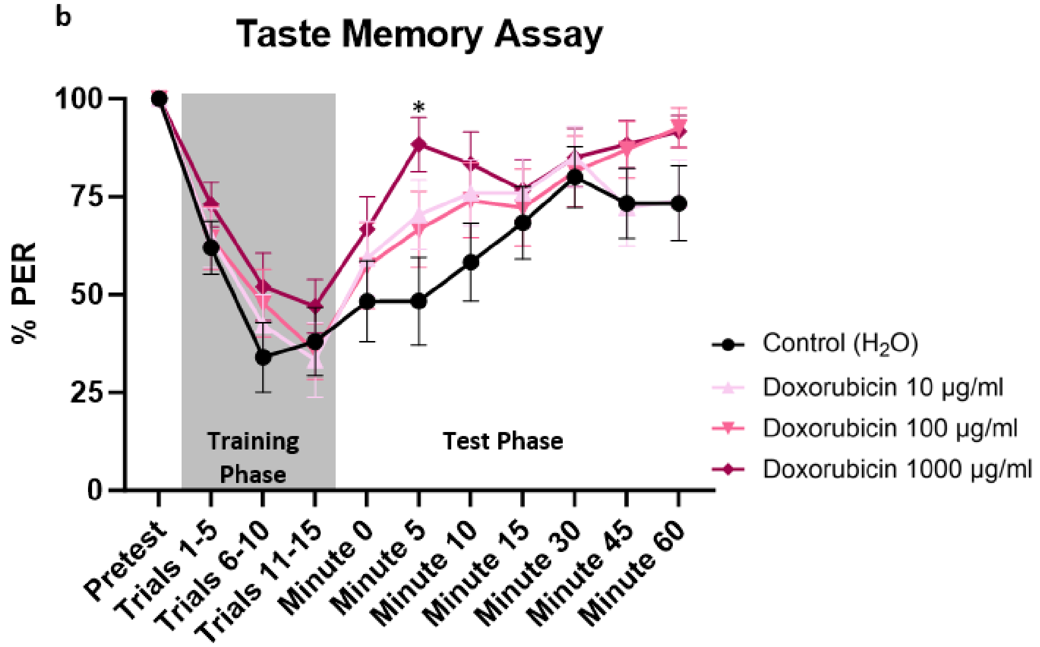

Performance of chemotherapy-treated *Drosophila* on the taste memory assay. There are trends towards impaired memory for cyclophosphamide- (**a**) and doxorubicin-treated flies (**b**), reaching statistical significance for the high dose doxorubicin group at minute 5 of the test phase. To better visualize the data, the graphs were separated by chemotherapeutic agent, but data for the vehicle control cohort are the same. Data are represented as the mean ± SEM. Statistical analysis was performed using the repeated measures 2-way ANOVA with Dunnett's multiple comparison test. Each cohort consists of n≥15 flies at 20 days post-eclosion. The genotype is w^{1118}. *p<0.05.
PER, proboscis extension reflex.

Supplemental Figure 3

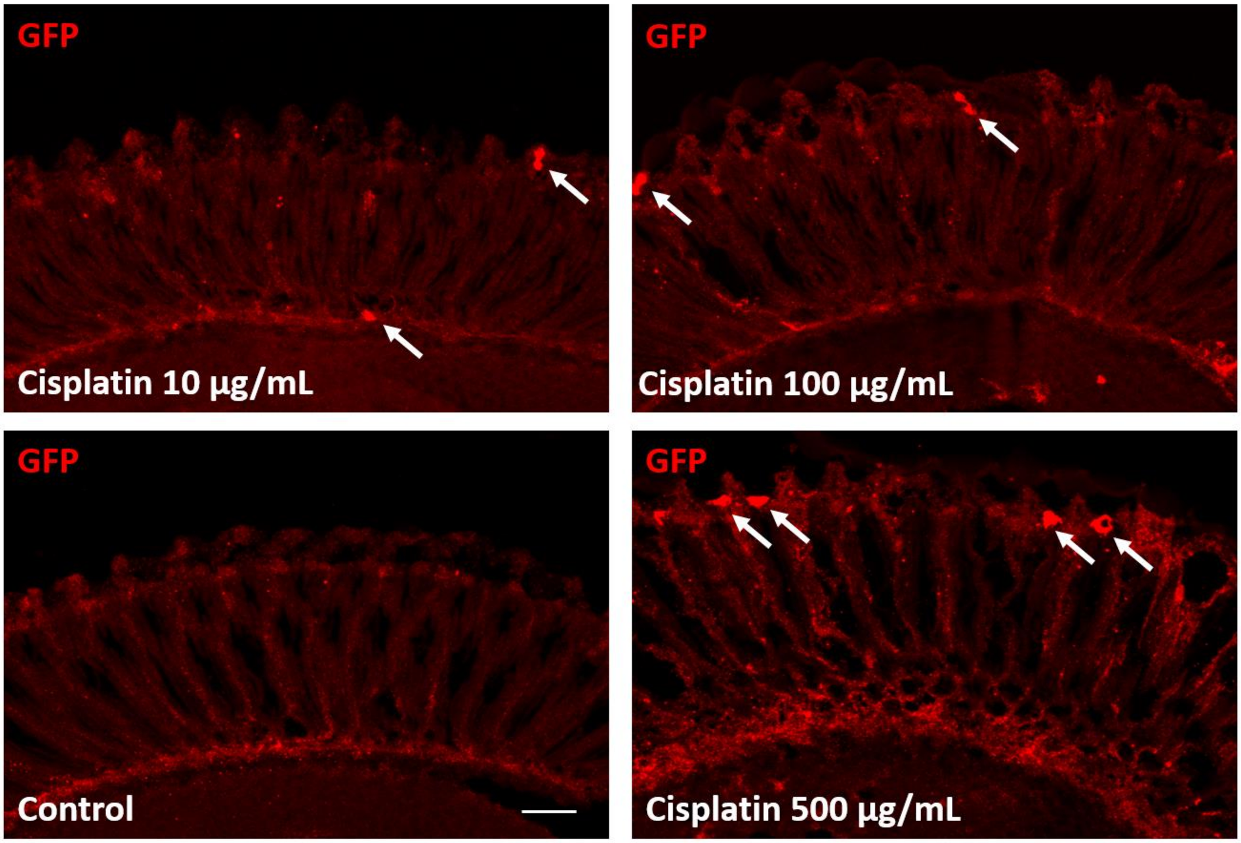

Oxidative stress in cisplatin-treated *Drosophila*, as assessed by *GstD1-GFP* activation. Immunofluorescence images of retina from cisplatin and control flies at day 30 are shown. Arrows highlight representative cells with *GstD1-GFP* activation, indicated by GFP positivity; scale bar, 20 µm. The genotype is *GstD1-GFP/+*.

Supplemental Table 1

	Percent Survival		
**Treatment**	**Day 10**	**Day 20**	**Day 30**
Control (H_2O)	98.9%	93.6%	83.3%
Cisplatin 10 µg/mL	98.0%	98.0%	98.0%
Cisplatin 100 µg/mL	99.0%	88.4%	67.6%
Cisplatin 500 µg/mL	92.4%	64.7%	61.0%
Cyclophosphamide 10 µg/mL	99.0%	97.9%	84.8%
Cyclophosphamide 100 µg/mL	97.9%	94.7%	76.6%
Cyclophosphamide 1000 µg/mL	96.8%	95.7%	64.0%
Doxorubicin 10 µg/mL	100%	97.9%	95.1%
Doxorubicin 100 µg/mL	88.0%	84.0%	71.8%
Doxorubicin 1000 µg/mL	92.8%	85.0%	71.0%

Survival data for chemotherapy and vehicle control cohorts at day 10, 20, and 30. Each cohort started with 86-105 w^{1118} flies.
